# Corrective Osteotomies in Severe Non-Idiopathic Lower Limb Alignment Disorders in the Aspect of Future Joint Endoprosthesis—A Perspective Study

**DOI:** 10.3390/jcm12196380

**Published:** 2023-10-06

**Authors:** Kamil Kołodziejczyk, Michał Saganek, Adam Czwojdziński, Rafał Garlewicz, Marcin Złotorowicz, Jarosław Czubak

**Affiliations:** Department of Replantation and Reconstruction, Centre of Postgraduate Medical Education, Professor A. Gruca Teaching Hospital, Konarskiego 13, 05-400 Otwock, Poland

**Keywords:** corrective osteotomies, lower limb malalignment, joint preservation, limb reconstruction

## Abstract

The aim of this study was to retrospectively evaluate the effectiveness of corrective osteotomies in lower limb axis disorders at different levels of non-idiopathic (post traumatic, developmental, post-septic) etiology. A total of 50 patients were divided into three groups: A—thigh segment alignment disorder (24 patients); B—tibia segment alignment disorder (18 patients); C—thigh and tibia segment alignment disorder (8 patients). Radiological evaluation of digital lower limb postural X-ray was performed laterally and for AP, and included mLPFA, mLDFA, MAD, CORA coronal and sagittal plane parameters for the femur segment and mMPTA, mLDTA, MAD, CORA coronal and sagittal plane for the tibia segment. Clinical assessment was based on the LLFI. The mean follow-up was 55.8 months (12–86). Improvements in the radiological parameters and statistical significance were achieved for all measurements in all groups (*p* < 0.05). The most common plane of deformation was the coronal plane (varus/valgus), followed by the transverse (rotational) and sagittal planes (procurvatum/recurvatum). In this study, we examined 29 post-traumatic deformities and 21 other etiologies. Improvements in the LLFI score performance after corrective osteotomies were observed in all three groups. Corrective osteotomies are a safe and useful but challenging method of preserving joints in cases of post-traumatic, developmental or post-septic lower limb alignment disorders.

## 1. Introduction

**Figure 1:**
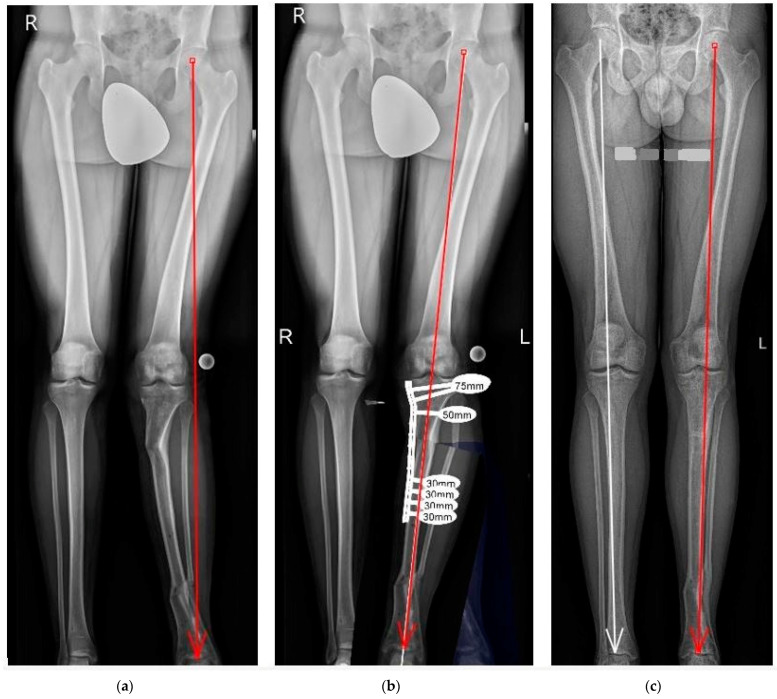
A 28-year-old man with post-traumatic valgus deformity of the left lower limb at the level of the proximal tibia: (**a**) preoperative standing AP and lateral X-ray, tibia valgus deformity of mMPTA: 92.5 degrees; mLDTA: 80.5 degrees; CORA coronal:—15 degrees; MAD:—62 mm; (**b**) preoperative planning medial close-wedge PTO with LCP plate (Stryker AxSOS, Kalamazoo, MI, USA); (**c**) postoperative bone fusion and normal axis of the lower limb.

**Figure 2:**
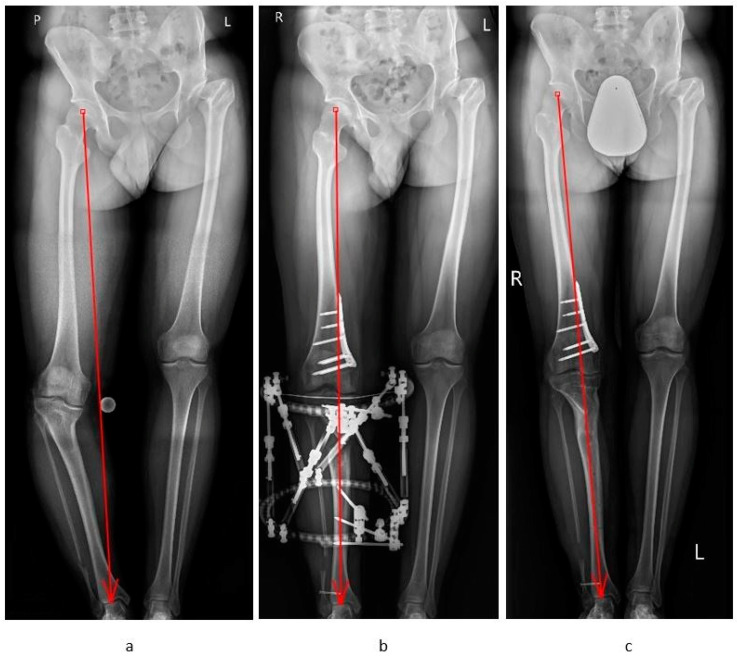
A 31-year-old man with post-septic complex deformity of the right lower limb at the distal femur valgus and varus proximal tibia: (**a**) preoperative standing AP and lateral X-ray; mLPFA: 64 degrees; mLDFA: 80.5 degrees; CORA coronal:—13 degrees, and the proximal tibia varus mMPTA: 67.5 degrees; mLDTA: 105 degrees; CORA coronal: 32 degrees; external torsion: 15 degrees; shortening: 3 cm; MAD: 86 mm; (**b**) operative treatment with medial close wedge DFO with LCP plate (DePuy Synthes TomoFix, Zuchwil, Switzerland) and tibia distraction osteotomy with TSF (Taylor Spatial Frame; Smith and Nephew, Memphis, TN, USA); (**c**) postoperative bone fusion and normal axis of the lower limb.

**Figure 3:**
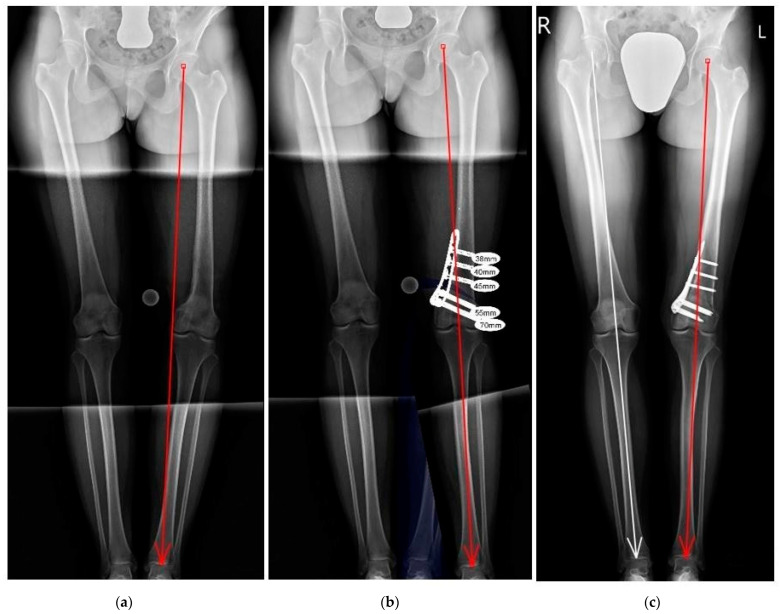
A 30-year-old woman with varus deformity of the left lower limb at the distal femur after resection of an aneurysmal tumor cyst in adolescence: (**a**) preoperative standing AP and lateral X-ray, varus deformity of the distal femur mLPFA: 95 degrees; mLDFA: 110 degrees; CORA coronal: 22 degrees; MAD: 46 mm; (**b**) preoperative planning corrective medial open wedge DFO with LCP plate (DePuy Synthes TomoFix, Zuchwil, Switzerland); (**c**) postoperative bone fusion and normal axis of the lower limb.

**Figure 4:**
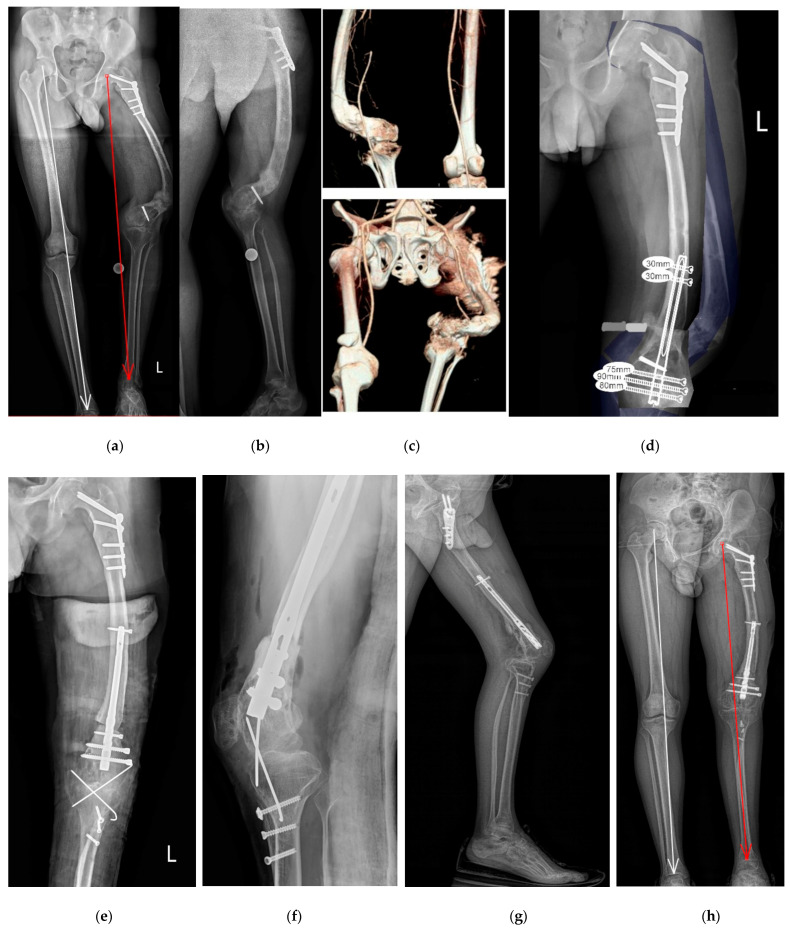
A 24-year-old man with congenital diseases of the femur and postoperative (severe surgical treatment outside our center) complex deformation of the left lower limb at the level of the femur; varus: 63 degrees; anteflexion: 64 degrees; internal torsion: 55 degrees; shortening: 4 cm; MAD: 50 mm; (**a**,**b**) preoperative radiograph standing AP and lateral; (**c**) image of deformity in 3D CT reconstruction; (**d**) preoperative planning; (**e**,**f**) postoperative X-ray: corrective osteotomy of the distal femoral and tibial tuberosity to reconstruct the patella traction of the knee, intramedullary femoral nail (ChM retrograde femoral nail, Lewickie, Poland) and screw fixation, osteotomy of the tibial tuberosity, temporary fixation of the knee joint with K-wires (10 days); (**g**,**h**) postoperative radiograph standing AP and lateral of the lower limb normal alignment.

Limb alignment disorders cause pain, discomfort and gradual loss of limb function in patients. If left untreated, or treated incorrectly, they can lead to degenerative changes, necessitating arthroplasty in the future. An osteotomy is an operation that allows us to preserve the joint. The aim of this type of treatment is to improve the alignment of the limb, relieve the appropriate joint compartment, reduce pain and slow the progression of degenerative changes [1]. Osteotomies have proven to be effective even in patients with severe osteoarthritis [2]. For example, patient satisfaction after osteotomies performed around the knee is higher than that after unicompartmental knee arthroplasty [3]. To achieve good clinical results, appropriate qualification, preoperative planning, a carefully performed procedure and postoperative rehabilitation are crucial [1]. Preoperative planning includes obtaining full-length standing radiographs of the limbs in anteroposterior (AP) and lateral projections, appointing physiological axes and angles of the lower limb and identifying deformities [4]. There are many studies on corrective osteotomies that have been used to treat degenerative idiopathic changes. However, there are few studies about osteotomies performed in non-idiopathic cases. Various factors or conditions can lead to lower limb alignment disorders, including injuries, metabolic bone diseases, septic shock and congenital and growth retardation in childhood. Our study showcases the treatment outcomes of patients with non-idiopathic disorders related to the alignment of the lower limb after osteotomy (Figure 1, Figure 2, Figure 3 and Figure 4).

## 2. Materials and Methods

This study was approved by the institutional review board (protocol number: 41/2021). Informed consent was obtained from all participants. 

The retrospective study group consisted of patients with lower limb axis disorders who qualified for surgical treatment between 2017 and 2022 using the corrective osteotomy method in our department. Mean follow-up was 55.8 months (12–86). The study included 50 patients (13 women and 37 men). The mean age was 45 years (18–86 years). The left side was affected in 21 patients and the right side was affected in 29 patients. The patients were clinically divided into three groups (Table 1).

Group A—thigh segment alignment disorder (24 patients): post traumatic 15, other 9 (fibrous dysplasia 3, congenital femur diseases 2, fibular hemimelia 2, post septic 2);

Group B—tibia segment alignment disorder (18 patients): post traumatic 14, other 4 (fibular hemimelia 3, Ollier disease 1);

Group C—thigh and tibia segment alignment disorder (8 patients): other 8 (Blount diseases 6, fibular hemimelia 1, post septic 2).

The classification of patients for radiological evaluation was based on the deformity within the bone segment of the extremity. The difficulty in evaluating the results of radiologic measurements involves the equalization of the results of these measurements of deformity in the frontal (valgus–varus) and sagittal (anterior and posterior flexion) planes. When deformity occurs at two levels (thigh, tibia), sometimes the MAD remains within the range of normal values and each segment is disturbed (valgus, varus), which may result in oblique alignment of the knee joint line and clinically patellofemoral and all other joint complaints.

Radiological evaluation was performed on a standard digital anteroposterior and lateral standing radiographs of the lower limb before the surgery and at the final follow-up visit to the orthopedic outpatient clinic. Radiographic assessment standing X-rays allowed the following angles to be measured: for femur segment: mLPFA (mechanical lateral proximal femoral angle), mLDFA (mechanical lateral distal femoral angle), MAD (mechanical axis deviation) and CORA (center of rotation of angulation) coronal and sagittal plane; for tibia segment: mMPTA (mechanical medial proximal tibial angle), mLDTA (mechanical lateral distal tibial angle), MAD (mechanical axis deviation) and CORA (center of rotation of angulation) coronal and sagittal plane [5]. Preoperative diagnosis and planning in patients with suspected torsional disorders were guided by computed tomography (CT) images with 3D and multiplanar reconstruction (MPR) [6]. All measurements were performed, accounted for and calculated statistically, considering the lower limb axis disorders relative to the segment. The known results were related to the normal values described by Paley [5]. Clinical assessment using the LLFI scale was performed together with radiological evaluation in our department just prior to surgery by the admitting physician and after surgery at the last follow-up in the outpatient clinic by a designated physician from our department. 

Statistical analysis used the Stata 11.0 software and Microsoft Excel. The Shapiro–Wilk tests did not show normal distributions for all variables and the Wilcoxon signed-rank test was used. The accuracy of the measurement was 0.5 degrees and 0.5 mm (all parameters were measured with CareStream Health, Rochester, NY, USA). The statistical significance level was set at *p* < 0.05.

## 3. Results 

The analysis of the pre- and postoperative results in patients with varus deformity on the femur segment showed that the change in LPFA, CORA coronal and MAD was statistically significant (*p* < 0.05). This indicates that for this disorder, the main correction is due to the improvement in the proximal part of the femur (Table 2).

For patients with valgus deformity on the femur, a satisfactorily significant change was observed in mLDFA, CORA coronal and MAD (*p* < 0.05). As the analysis shows, in the operation for a valgus disorder on the femur, the correction mainly concerns the distal segment (Table 3). 

In patients with tibial varus deformity, statistically significant correction was observed for mMPTA, CORA coronal and MAD (*p* < 0.05). As observed for the same disorder on the femur, the correction of the axis results from the restoration of the correct angular values in the proximal segment of the tibia (Table 4). 

In patients with tibial valgus deformity, there was no statistically significant improvement in mMPTA (*p* = 0.11) and mLDTA (*p* = 0.103). Nevertheless, in these patients, a satisfactorily significant improvement in CORA coronal and MAD (*p* < 0.05) was obtained. These parameters best illustrate the axis of the limb in the frontal plane (Table 5).

A comparative analysis was also performed for the three groups, comparing the groups with one other in terms of demographic and operational data. Statistically significant differences in the time to bone union were observed between groups B versus C and A versus C (Table 6). In group C (corrective osteotomy on two segments) the time to bone union was significantly shorter than in groups A and B (Table 1).

In the clinical analysis of the main three groups of patients, statistically significant differences were obtained between pre- and postoperative LLFI scale scores (Table 7).

The knowledge of the values of the LLFI scale scores for individual patients allowed a statistical comparative analysis within each deformity for a given segment similar to the analysis of radiological measurements. Analyzing the clinical LLFI scale scores within a segment revealed statistically significantly worse preoperative scores for the varus deformity for both the thigh (*p* = 0.02) and tibia (*p* = 0.04) segments. The postoperative LLFI scale scores in the comparative analysis of deformity within one segment showed no statistical significance (Table 8).

The clinical comparison of deformities within the two segments revealed lower LLFI scale values prior to surgery in the varus (*p* = 0.04) and valgus (*p* = 0.052) deformities at the thigh level compared to the tibia segment. The postoperative LLFI scale scores in the comparative analysis of varus/valgus deformity showed no statistical significance (Table 9).

This analysis indicates that the greater problem in daily life is the varus deformity in both the thigh and tibia deformities. 

Deformities in the sagittal plane, rotational disorders and accompanying limb shortening were not subjected to detailed statistical analysis due to insufficient data. The predominant lower limb axis disorder remains the frontal plane, which may also be the least favorable for compensation.

In the material presented here, none of the patients required conversion to a hip, knee or ankle endoprosthesis. Statistically significant differences in preoperative and postoperative LLFI scale values indicate the very high value of corrective osteotomy treatment of lower extremity deformities of diverse localization and etiology.

We observed four complications in the study material. In group A, we noted one loosening fixation and fusion in abnormal alignment without the need for surgical intervention due to the advanced age of the patient, and one non-union, which we treated with surgery using the Masquelet technique and achieved bony healing in the proper alignment. In Group B, on the other hand, we observed two infectious complications that required surgical treatment; we also achieved bony healing in the proper alignment.

## 4. Discussion

Corrective osteotomy is a complex surgical procedure that requires the surgeon to have a great deal of knowledge and experience in reconstructive surgery. The outcome of any corrective osteotomy is affected by two main factors. The first is proper planning, which allows the possibility of achieving the expected correction to be determined, and the second factor is that the operation must be performed correctly to achieve the intended result. The goal of this surgical technique is to suppress, delay and prevent the development of osteoarthritis, potentially concluding with the replacement of the joint with its endoprosthesis.

In our clinic, we perform X-rays of both limbs and we determine angles according to Paley’s classification. Preoperative planning is a key part of treatment. Proper measurement of the angles, slopes and correction are helpful, allowing adjustment of the hinge axis position and reducing the risk of an opposite cortical hinge fracture [7]. Computer assistance, especially 3D and multiplanar reconstructions, help us to visualize deformities, plan corrections and achieve stable anastomosis of bone fragments. It can improve the accuracy and precision of postoperative coronal and sagittal alignments and improve postoperative results with decreased radiation exposure [8]. Iorio et al. showed that patients who receive computer-assisted HTO have a much higher probability of correction (86%) compared to patients with conventional HTO (23%) [9]. However, this technique has some disadvantages. This solution is not available in all hospitals, and has a long learning curve and notable line of sight issues [10]. Computer-assisted treatment methods are becoming increasingly popular and helpful in complicated cases, such as that of genu recurvatum. Bakircioglu et al. showed that a computer-assisted hexapod external fixator provides high-precision multiplanar correction of the deformity. After the procedure, the patellar alignment remains stable [11]. It is mandatory to relieve the loaded compartment after osteotomy. Proper correction is necessary in both the sagittal and coronal plane. During the HTO, it is recommended that the posterior tibial slope remain unaltered. The posterior tibial slope increases in anteriorly inclined osteotomy and decreases in posteriorly inclined osteotomy. The change in the posterior tibial slope is proportionally related to the absolute value of the osteotomy inclination angle [12].

The choice of the appropriate treatment depends on the deformity and the planned correction. Medial open-wedge (MOW) tibial osteotomy is generally performed in patients with medial compartment osteoarthritis. This type of treatment can cause tibial slope changes, medial collateral tightening and patella baja. On the other hand, lateral close-wedge (LCW) tibial osteotomy can lead to overhang of the tibial plateau, producing changes in tibio-condylar offset [13]. Overcorrection during HTO of a medial proximal tibial angle of >95° leads to increased shear stress on the articular cartilage and consequent inferior clinical outcomes [14]. Nejima et al. documented that an increased joint line convergence angle (JLCA) and decreased medial proximal tibial angle (mMPTA), which manifests as an oblique joint line, were risk factors for overcorrection of mMPTA. The authors note that the possibility of this complication in the above deformity is as high as 40% [15]. Other authors have reported that combining high tibial osteotomy with valgus creation (CWO) is expected to incorporate the advantages of both LCW and MOW techniques for HTO, reducing the risk of the above-mentioned complication. This modification avoids metaphyseal tibial bone loss, decreasing transposition of the tibial condyle and shortening of the patellar tendon after osteotomy, even in cases of significant correction [16]. Arthritis of the patellofemoral joint is a contraindication for the classic osteotomy around the knee [17]. Patients with a patellofemoral joint compartment have the highest complication rates after total knee replacement, and thus require appropriate treatment [18]. HTO increases pressure and stress on the anterior knee joint sector. Kloos et al. considered that a biplanar distal osteotomy led to a significant decrease in pressure in the patellofemoral joint in a biomechanical study [19]. 

Their study has limitations, including a relatively short follow-up and small patient population, as well as heterogeneous deformities that are very difficult to define, describe and compare due to their varied etiology, complexity and multilevel lower limb deformities.

In our study, we have chosen various methods of stabilization after osteotomy. The choice of treatment method depends on the type of operation which will be performed. Intramedullary (IM) nailing for deformity correction is a better option, because it has the advantage of both an external fixator and an internal fixator [20]. In IM fixation, the contact surface in the osteotomy line increases, which provides a shorter healing time compared to other methods [21]. Pietrzak et al. demonstrated that IM nailing is the best option for femoral length disorder compared to a monoliteral external distractor and external fixator [22]. Antegrade IM femoral nailing with distal hemiepiphysiodesis is a very promising therapeutic option when young patients require correction of deformity and limb lengthening [23]. It was also proved that healing parameters in older populations are similar to those of younger patients [24]. Failures after IM fixations are rare but immensely challenging. In these cases, fixation with a longer implant may be the best option [25]. To achieve optimal results after the operation, new procedures during the operation are investigated. It is proved that using the K-wires for protection and stabilization can improve the lateral hinges’ resistance to failing during the opening of the osteotomy. This can be achieved by placing a K-wire at the theoretical location of the lateral hinge [26]. Filling the gap after osteotomy remains controversial. Defects larger than 10 mm can be filled with bone auto- or allografts. In many studies, the risk of non-union and complications was comparable in the group with and without grafts [27]. In our practice, we have used bone allografts to perform open-wedge corrective osteotomies of more than 10 mm and have observed only two cases of destabilization or lack of adhesions. Novel studies are aiming to investigate the best material for bone graft. Using the reamer–irrigator–aspirator system and collecting the ipsilateral bone graft from the femur may be a promising option in such cases [28]. 

Based on this analysis, we can conclude that varus deformity, both in situations of deformity of the thigh and tibia, is a significant problem in everyday life. Statistically significant differences in the pre- and postoperative LLFI scale values in all three groups (Table 7) indicate that corrective osteotomy is a highly effective treatment for lower limb deformities of various locations and etiologies. Osteotomies around the knee have an excellent success rate, but some patients will require total knee arthroplasty in the future. To preserve as much of the joint as possible through corrective osteotomy and the correct alignment of the limb, it will be possible to perform a unicompartmental knee arthroplasty in the future [29,30]. None of our patient requires knee arthroplasty at this time; however, most of them remain in the observation period and it cannot be ruled out that some of them will require joint replacement in the future.

Osteotomies are well-known methods of treatment and joint preservation. Our results showed that a properly performed procedure can significantly improve the patient’s quality of life. This is in agreement with the findings from recent studies. Patients who have received a double-level osteotomy around the knee have better results both in the International Knee Documentation Committee Subjective Knee Form and the Knee injury and Osteoarthritis Outcome Score [31].

## 5. Conclusions

The treatment of patients with lower limb malalignment is highly complicated from the planning level to surgical treatment. These patients are usually young and require conservative joint treatment. In our study, we showed that corrective osteotomies are a great therapeutic option without joint replacement with an endoprosthesis in such cases. Proper preoperative planning and the selection of an appropriate stabilization method can lead to very good clinical results. However, indications for this procedure should be cautious in patients with coexisting diseases, and should involve careful consideration of their individual risk factors. Ultimately, the choice of treatment method depends on the experience and possibilities of the surgeon.

## Figures and Tables

**Table 1:** Demographic data of all three groups of patients.

	GROUP A—Femur Segment Alignment Disorder	GROUP B—Tibia Segment Alignment Disorder	GROUP C—Femur and Tibia Segment Alignment Disorder	TOTAL
Mean age (year)	47 (18–86)	44 (18–72)	38 (18–52)	45 (18–86)
Sex	5 Female 19 Male	4 Female 14 Male	4 Female 4 Male	13 Female 37 Male
Patient number	24	18	8	50
Operation side	13 Right 11 Left	12 Right 6 Left	4 Right 4 Left	29 Right 21 Left
Etiology	Post-traumatic 15; Other 9: Fibrous dysplasia 3, Congenital femur diseases 2, Fibular hemimelia 2, Post septic 2	Post-traumatic 14; Other 4: Fibular hemimelia 3, Ollier disease 1	Other 8: Blount diseases 6, Fibular hemimelia 1, Post septic 2	Post-traumatic: 29; Other: 21
Deformity	Varus: 20 Valgus: 3 Torsion: 11 Shortening: 1 Sagittal: 7	Varus: 8 Valgus: 8 Torsion: 9 Shortening: 3 Sagittal: 6	Varus: 7 Valgus: 8 Shortening: 1	Varus: 35 Valgus: 19 Torsion: 20 Shortening: 5 Sagittal: 13
Osteotomy level	PFO: 9 Femur shaft: 6 DFO: 9	PTO: 6 Tibia shaft: 3 DTO: 9	DFO: 7 PTO: 8 Femur shaft: 1	PFO: 9 Shaft f: 7 DFO: 16 PTO: 14 Shaft t: 3 DTO: 9
Osteosynthesis	LCP: 10 Intramedullary nail: 15	LCP: 6 Intramedullary nail: 11 TSF: 2	LCP: 10 Intramedullary nail: 1 TSF: 3	LCP: 26 Intramedullary nail: 27 TSF: 5
Deformation duration (mean months)	150.7 (±103.5)	184.5 (±76.4)	200.6 (±60.3)	170.7 (±89.5)
Operative time (mean minutes)	162 (±61.9)	156.3 (±52.9)	158.7 (±16.4)	159.4 (±53.2)
Time to bone union (mean months)	7.44 (±3.5)	7.05 (±2.5)	4.1 (±0.84)	6.7 (±3.11)
Complication:	2 (Destabilization, non-union)	2 (Infection)	0	4
Follow-up (mean months)	53.5 (±18.7)	57.8 (±15.7)	58 (±19.8)	55.8 (±17.6)

LCP—locking low-contact plate; TSF—Taylor Spatial Frame; PFO—proximal femur osteotomy; DFO—distal femur osteotomy; PTO—proximal tibia osteotomy; DTO—distal tibia osteotomy.

**Table 2:** Radiological measurement results of varus femoral segment disorder.

Femur Varus Alignment Disorders (Mean) *n* = 22	Pre-Operation SD (95% CI)	Post-Operation SD (95% CI)	Diff.	*p*-Value
mLPFA (80–90 deg)	102.5 ± 11.5 (90–127.5)	91.5 ± 5.3 (80–102)	−11.0	<0.005
mLDFA (85–90 deg)	94.5 ± 8.7 (81.5–117)	90.0 ± 1.7 (87–93)	−4.5	0.01
CORA coronal	29.5 ± 19.7 (11.5–80.5)	5.0 ± 9.0 (0–36.0)	−24.5	<0.005
CORA sagittal	8.0 ± 16.5 (0–64.0)	1.0 ± 4.0 (0–20.0)	−7.0	0.03
MAD (10–15 mm)	26.5 ± 17.0 (5.0–65.0)	7.0 ± 8.0 (0–28.0)	−19.5	<0.005

mLPFA—mechanical lateral proximal femoral angle; mLDFA—mechanical lateral distal femoral angle; CORA—center of rotation of angulation; MAD—mechanical axis deviation; Diff—difference; *p*-value Wilcoxon signed-rank test.

**Table 3:** Radiological measurement results of valgus femoral segment disorder.

Femur Valgus Alignment Disorders (Mean) *n* = 10	Pre-Operation SD (95% CI)	Post-Operation SD (95% CI)	Diff.	*p*-Value
mLPFA (85–90 deg)	81.0 ± 7.62 (64.0–89.0)	84.0 ± 8.0 (67.5–89)	3.0	0.18
mLDFA (85–90 deg)	75.5 ± 4.6 (67.0–81.0)	87.5 ± 2.63 (83.5–91.5)	12.0	<0.005
CORA coronal	−12.5 ± 4.5 (−7.5–−20.0)	0.05 ± 1.82 (−2.5–2.5)	12.55	<0.005
CORA sagittal	0.0	0.0	0	1
MAD (10–15 mm)	−26.5 ± 8.1 (−16.0–−40.0)	4.5 ± 7.3 (−6.0–15.0)	31.0	<0.005

mLPFA—mechanical lateral proximal femoral angle; mLDFA—mechanical lateral distal femoral angle; CORA—center of rotation of angulation; MAD—mechanical axis deviation; Diff—difference; *p*-value Wilcoxon signed-rank test.

**Table 4:** Radiological measurement results of varus tibia segment disorder.

Tibia Varus Alignment Disorders (Mean) *n* = 14	Pre-Operation SD (95% CI)	Post-Operation SD (95% CI)	Diff.	*p*-Value
mMPTA (85–90 deg)	78.5 ± 9.1 (63.0–89.5)	88.0 ± 2.5 (82.5–90.0)	9.5	<0.005
mLDTA (86–92 deg)	100.5 ± 14.1 (76.0–124.0)	92.0 ± 4.2 (89.0–99.0)	−8.5	0.02
CORA coronal	22.5 ± 7.7 (10.0–33.0)	2.0 ± 2.8 (0–6.5)	−20.5	<0.005
CORA sagittal	9.5 ± 13.3 (0–40)	1.5 ± 4.6 (0–15.5)	−8.0	0.02
MAD (10–15 mm)	40.5 ± 20.7 (22.0–95.0)	9.5 ± 6.24 (2.0–20.0)	−31.0	<0.005

mMPTA—mechanical medial proximal tibial angle; mLDTA—mechanical lateral distal tibial angle; CORA—center of rotation of angulation; MAD—mechanical axis deviation; Diff—difference; *p*-value Wilcoxon signed-rank test.

**Table 5:** Radiological measurement results of valgus tibia segment disorder.

Tibia Valgus Alignment Disorders (Mean) *n* = 10	Pre-Operation SD (95% CI)	Post-Operation SD (95% CI)	Diff.	*p*-Value
mMPTA (85–90 deg)	92.0 ± 5.4 (81.5–101.0)	89.5 ± 3.1 (86.0–96.5)	−2.5	0.11
mLDTA (86–92 deg)	82.0 ± 8.2 (65.5–93.0)	86.0 ± 4.8 (78.0–93.0)	4.0	0.103
CORA coronal	−14.0 ± 4.9 (−8.0–−21.0)	−0.5 ± 1.9 (−4.2–3.0)	13.5	<0.005
CORA sagittal	5.0 ± 14.5 (−12.0–36.5)	1.5 ± 5.2 (−8.0–12.0)	−3.5	0.26
MAD (10–15 mm)	−25.0 ± 6.7 (−34.0–−17.0)	3.0 ± 5.4 (−5.0–10.0)	28.0	<0.005

mMPTA—mechanical medial proximal tibial angle; mLDTA—mechanical lateral distal tibial angle; CORA—center of rotation of angulation; MAD—mechanical axis deviation; Diff—difference; *p*-value Wilcoxon signed-rank test.

**Table 6:** Demographic and operation data comparison of the main three groups of patients.

Patient Group Comparison	Deformation Duration (mth) (*p*-Value)	Operative Time (min) (*p*-Value)	Time to Bone Union (mth) (*p*-Value)	Follow-Up (mth) (*p*-Value)
Group A/B	150.7 ± 103.5/184.5 ± 76.4 (*p* = 0.110)	162 ± 61.9/156.3 ± 52.9 (*p* = 0.372)	7.44 ± 3.5/7.05 ± 2.5 (*p* = 0.337)	53.5 ± 18.7/57.8 ± 15.7 (*p* = 0.202)
Group B/C	184.5 ± 76.4/200.6 ± 60.3 (*p* = 0.283)	156.3 ± 52.9/158.7 ± 16.4 (*p* = 0.429)	7.05 ± 2.5/4.1 ± 0.84 (*p* < 0.005)	57.8 ± 15.7/58 ± 19.8 (*p* = 0.494)
Group A/C	150.7 ± 103.5/200.6 ± 60.3 (*p* = 0.054)	162 ± 61.9/158.7 ±16.4 (*p* = 0.406)	7.44 ± 3.5/4.1 ± 0.84 (*p* < 0.005)	53.5 ± 18.7/58 ± 19.8 (*p* = 0.292)

mth month; min minutes; *p*-value Wilcoxon signed-rank test.

**Table 7:** LLFI clinical results for each patient group.

LLFI (Mean)	Pre-Operation SD (95% CI)	Post-Operation SD (95% CI)	Diff.	*p*-Value
Group A	31.8 ± 4.8 (24.0–42.0)	72.3 ± 5.8 (55.0–80.0)	40.5	<0.005
Group B	39.4 ± 9.5 (25.0–58.0)	70.1 ± 6.8 (59.0–79.0)	30.67	<0.005
Group C	36.7 ± 5.4 (30.0–45.0)	74 ± 3.6 (69.0–79.0)	37.25	<0.005

Diff—difference; LLFI—Lower Limb Functional Index; *p*-value Wilcoxon signed-rank test.

**Table 8:** Comparative analysis of LLFI clinical results within a single segment deformity.

LLFI (Mean)	Varus Femur *n* = 22	Valgus Femur *n* = 10	Diff.	*p*-Value
Pre-operation SD (95% CI)	32.0 ± 5.5 (24.0–45.0)	35.8 ± 4.6 (30.0–42.0)	3.8	0.02
Post-operation SD (95% CI)	72.5 ± 6.0 (55.0–80.0)	73.8 ± 3.99 (68.0–79.0)	1.3	0.28
	**Varus Tibia *n* = 14**	**Valgus Tibia *n* = 10**		
Pre-operation SD (95% CI)	35.3 ± 5.8 (25.0–46.0)	42.0 ± 10.3 (25.0–45.0)	6.7	0.04
Post-operation SD (95% CI)	71.7 ± 5.6 (63.0–79.0)	71 ± 7.8 (59.0–79.0)	0.7	0.37

Diff—difference; LLFI—Lower Limb Functional Index; *p*-value Wilcoxon signed-rank test.

**Table 9:** Comparative analysis of LLFI clinical scores according to the type of deformity and the segment involved.

LLFI (Mean)	Varus Femur *n* = 22	Varus Tibia *n* = 14	Diff.	*p*-Value
Pre-operation SD (95% CI)	32.0 ± 5.5 (24.0–45.0)	35.3 ± 5.8 (25.0–46.0)	3.3	0.04
Post-operation SD (95% CI)	72.5 ± 6.0 (55.0–80.0)	71.7 ± 5.6 (63.0–79.0)	0.8	0.3
	**Valgus Femur *n* = 10**	**Valgus Tibia *n* = 10**		
Pre-operation SD (95% CI)	35.8 ± 4.6 (30.0–42.0)	42.0 ± 10.3 (25.0–45.0)	6.2	0.052
Post-operation SD (95% CI)	73.8 ± 3.99 (68.0–79.0)	71 ± 7.8 (59.0–79.0)	2.8	0.14

Diff—difference; LLFI—Lower Limb Functional Index; *p*-value Wilcoxon signed-rank test.

## Data Availability

All data generated or analyzed during this study are included in the published article.

## Abbreviations

DFO	distal femur osteotomy
HTO	high tibia osteotomy
PTO	proximal tibia osteotomy
MOW	medial open wedge
LCW	lateral close wedge
JLCA	joint line convergence angle
CWO	combined wedge osteotomy
TSF	Taylor Spatial Frame
LCP	locking plate
IM	intramedullary nail
AP	antero-posterior
